# Taisui TS-2007S, a Large Microbial Mat Discovered in Soil in China

**DOI:** 10.3389/fmicb.2020.592034

**Published:** 2020-11-11

**Authors:** Tongfu Su, Haohao Liu, Chaohui Zhang, Di Shang, Chaojiang Wang, Liyou Qiu

**Affiliations:** ^1^College of Sciences, Henan Agricultural University, Zhengzhou, China; ^2^Key Laboratory of Enzyme Engineering of Agricultural Microbiology, Ministry of Agriculture and Rural Affairs, College of Life Sciences, Henan Agricultural University, Zhengzhou, China; ^3^College of Life Science and Technology, Henan Institute of Science and Technology, Xinxiang, China; ^4^Institute of Genetics and Physiology, Hebei Academy of Agriculture and Forestry Sciences, Shijiazhuang, China

**Keywords:** microbial mats, extracellular polymeric substances, chemical composition, spectra analysis, microbial community structure, Taisui

## Abstract

In this study, Taisui TS-2007S, a previously unidentified biological object discovered in soil in China, was identified. TS-2007S was shown to contain abundant carbohydrates but a scarcity of protein, fat, and minerals. The exopolymers of TS-2007S showed FT-IR spectra that were similar to those of xanthan gum (XG) but that were dissimilar to those of polyvinyl alcohol (PVA). The NMR spectra of TS-2007S exopolymers in D_2_O were similar to those of PVA but differed from those of xanthan gum. Unlike PVA, TS-2007S exopolymers and xanthan gum were not soluble in dimethyl sulfoxide (DMSO). Furthermore, the exopolymers contained many monosaccharide components, including fucose, rhamnose, mannose, and glucuronic acid in a molar ratio of 87.90:7.49:4.45:0.15. The exopolymers also included traces of glucuronic acid, galactose, and xylose. Taken together, these results suggest that the exopolymers are microbial extracellular polymeric substances (EPSs). The microbial community structure in TS-2007S showed that the predominant bacterial, archaeal, and fungal phyla were Proteobacteria, Euryarchaeota, and Ascomycota at high relative abundances of 90.77, 97.15, and 87.43%, respectively, different from those observed in water and soil environments. Based on these results, we strongly propose that TS-2007S should be defined as a microbial mat formed in soil.

## Introduction

The ancient Chinese book *Shan Hai Jing*, published over 2000 years ago, and numerous later ancient books recorded meat-like objects excavated from the soil, and this type of object was named Taisui. In the past 30 years, there have been more than 200 recorded instances of Taisuis being unearthed from soil in many places in China (Wang and Wang, 2015). The surface texture of Taisui is hard, while the internal texture is soft and fleshy. These objects are irregular oblong, pointed cylinder, or flat pillow-like shape, are 30–150 cm in length and 10–60 cm in width, and are yellow, brown, white, or black in color. Taisuis have been regarded as ominous signs, but they have been used since ancient times in China to promote health and longevity (Zhao et al., 2018; Li et al., 2020). However, the biological characteristics of Taisuis have never been fully elucidated; thus, Taisuis are still named “unidentified biological objects.”

To elucidate the biological characteristics of Taisui, Huang and Dong studied the slices of a Taisui (unearthed from Zhouzhi, Shaanxi) and observed the presence of a variety of slime molds resided that they subsequently cultured. Thus, they considered that Taisui may be a slime mold complex (Huang and Dong, 1993). Zhu et al. (2011) showed that Taisuis (unearthed from Fuxin, Shenyang, and Tieling, Liaoning) contain polysaccharides, proteins, fats, nucleic acids, and mineral elements, but the protein content was much lower than that observed in microbial cells. Metagenomic analysis showed that Taisuis (unearthed from Qingdao, Shandong; Yili, Xinjiang; Sanmenxia, Henan) are rich in bacteria and archaea (Wang and Wang, 2017; Han et al., 2018). Surprisingly, Zhao et al. (2018) and Li et al. (2020) reported that the Fourier-transform infrared (FT-IR) spectroscopy and NMR spectra of Taisuis (unearthed from Zhungeer, Inner Mongolia; Beizhen, Liaoning; Yinchuan, Ningxia; Huangling, Shaanxi) are consistent with those of polyvinyl alcohol (PVA), and they speculated that Taisuis may be the construction wastes or PVA of nonbiological origin. The microbial community structure of Taisuis is similar to that detected in the environment, indicating that the microorganisms in the environment are simply embedded in PVA. Furthermore, Taisuis have been dated as being 40,000 years old by AMS-^14^C measurement.

Microbial mats are complex microbial communities visible to the naked eye that develop from biofilms on soil, rock, or aquatic sediment surfaces (Gerdes, 2010; Bolhuis et al., 2014). Microbial mats are ecosystems maintained by a matrix of exopolysaccharides that allows the convenient transmission of material, energy, and information among microbes and that promotes their survival in an extreme environment (Neu, 1994; Ruvindy et al., 2016; Prieto-Barajas et al., 2018). Microbial mats are potentially the oldest biological communities still flourishing today. The oldest microbial mats are 3.7 Ga-year-old stromatolites found in the Isua supracrustal belt (ISB) in Southwest Greenland (Nutman et al., 2016). Stromatolites are laminated structures formed by the deposition of microbial mat layers and are the most abundant fossils recognized from the Archaean and Proterozoic eras (Marais, 1990; Awramik, 1991). Ancient stromatolites are highly useful for studying the origin of life on early Earth (Baumgartner et al., 2020). The laminations are the result of the altered growth of phototrophs with different pigment compositions. Nevertheless, depending on environmental fluctuations, nonlaminated microbial mats can be found, for example, micrites, microbial mats formed in cavities on marine sediments, show a nonlaminated and strongly clotted texture (Esteve et al., 1992; Wood et al., 1993).

Microbial mats can be formed in a wide variety of environments in the biosphere, including streams, lakes, soils, and the ocean, as well as in extreme environments, including those with high or low temperature, solar (UV) irradiation, salinity or alkalinity conditions, or desiccation (Severin and Stal, 2010; Prieto-Barajas et al., 2018). The size of microbial mats varies from several millimeters to over 10 m in height and width, such as up to 4-m-high massive microbial mats that form at methane seeps in anoxic waters of the northwestern Black Sea shelf (Michaelis et al., 2002). In addition, widespread Archean and Proterozoic stromatolites range from millimeters to tens of meters in height and extend laterally from small lenses to aggregates hundreds of kilometers wide (Bosak et al., 2013).

Microbial mats are formed based on autotrophic metabolism (Stal et al., 2017). In illuminated environments, most bacterial mats are supported by Cyanobacteria that carry out oxygenic photosynthesis and atmospheric dinitrogen fixation, with these mats being called cyanobacterial mats (Stal, 2001). A few phototrophic microbial mats depend on anoxic photosynthesis by purple/green photosynthetic sulfur bacteria and are called anoxygenic microbial mats (Grant and Gust, 1987; van Gemerden et al., 1989; Castenholz et al., 1990). In environments where sunlight is absent and when reduced solutes or gases are available, chemolithoautotrophy mats are formed. Some chemolithoautotrophic mats are based on colorless sulfur bacteria that oxidize sulfide minerals to synthesize organic carbon, while others rely on methanotrophs and sulfate-reducing bacteria to oxidize H_2_S or methane to synthesize organic carbon (Konhauser, 2007).

Metagenomic studies using next-generation sequencing (NGS) technologies have revealed that abundant microbial diversity exists in microbial mats. The dominant microbes in microbial mats belong to the domain “Bacteria,” whereas the domain “Archaea” is dominant in the most extreme environments (Casamayor et al., 2002). The microbial diversity at higher taxonomic levels (phyla, class, and order) show fewer assemblages along more extreme environments, while specific bacteria species reveal the microdiversity of ecotypes (Melendrez et al., 2011). In addition to the essential bacterial phyla Cyanobacteria, Chlorobi (to which green sulfur bacteria belong), and Proteobacteria (to which purple and sulfate-reducing bacteria belong), Acidobacteria, Actinobacteria, Bacillariophyta, Bacteroidetes, Chloroflexi, Euryarchaeota, Firmicutes, Planctomycetes, Spirochaetes, and Verrucomicrobia are the most abundant phyla in bacterial mats (Guerrero et al., 2002; Prieto-Barajas et al., 2018). However, there are exceptions, such as with respect to heterotrophs, especially *Pseudomonas* species, being abundant in the microbial mats in a red desiccation pond located at the oligotrophic Cuatro Ciénegas Basin (Bonilla-Rosso et al., 2012; Peimbert et al., 2012).

Microbial mats can provide favorable reserves for biological remains and ephemeral biogenic features, including footprints, tracks, and other markings, and they play an important role in taphonomy (Gall, 2001). Microbial mats display a wide variety of metabolic processes and exhibit high proliferation in various extreme environments. Therefore, microbial mats are potential bioremediation systems and gene resources for industrial enzymes, antibiotics, and other biologically active substances (Prieto-Barajas et al., 2018).

In the present study, we analyzed the chemical composition and microbial communities of a Taisui excavated from Sanmenxia, central China. Our results strongly suggest that Taisuis should be considered microbial mats.

## Materials and Methods

### Samples and Reagents

**Figure 1:**
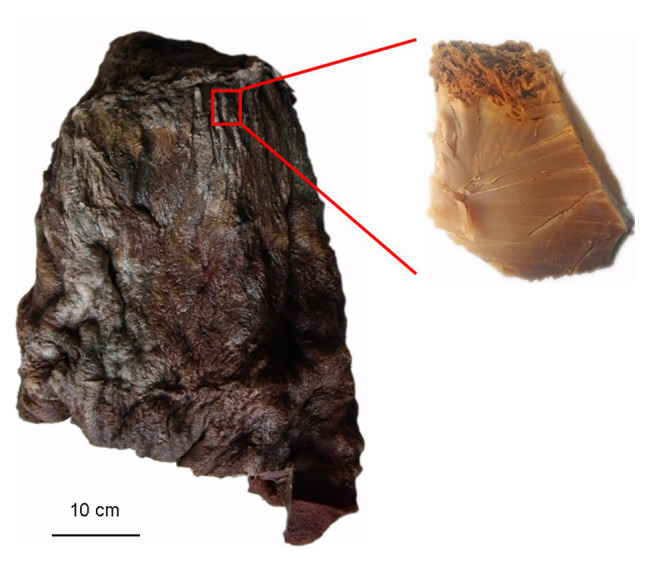
A photograph of the Taisui TS-2007S discovered on soil surface at Hubin District of Sanmenxia City, located south bank of the middle Yellow River in 2007, and a schematic diagram of sampling for studies in this research.

Taisui TS-2007S was discovered on the soil surface in Hubin district of Sanmenxia city, located on the south bank of the middle Yellow River, in 2007. TS-2007S weighed 20 kg and had a pointed cylinder shape and a tough texture with elasticity. The surface and internal tissue of TS-2007S was brown. The excavated peak of TS-2007S was 10 × 5 × 4 cm in length, width and height, respectively (Figure 1), and a 2–3 cm epidermal section removed. The inner layer was repeatedly rinsed with sterile saline and then sliced or collected as debris with a thickness of less than 1 mm. The slices or debris were added to distilled water at ratio of 1:10 and were dissolved in an 85°C water bath. The resulting solution was used for chemical composition analysis, or filtered using a 0.22-μm filter membrane. The filtrate was designated as exopolymers of TS-2007S.

Polyvinyl alcohol (analytically pure, average degree of polymerization 1,788 ± 50) and xanthan gum (XG; food grade, relative molecular mass over 1,000,000) were provided by Kelong Chemical Co., Ltd. (Chengdou, China), and Songguan Biotechnology Co., Ltd. (Nanjing, China), respectively.

### Chemical Composition Analysis

The protein, fat, ash, and moisture contents of the Taisui TS-2007S were determined following the Association of Official Analytical Chemists (AOAC) procedures (Association of Official Analytical Chemists, 2019). The total nitrogen content was determined using a Kjeltec™ 8,400 Auto Sampler System (FOSS, Hiller, Denmark) with 6.25 as a conversion factor. The fat content was determined by petroleum ether extraction in a Soxhlet apparatus. The ash content was determined by incineration in a muffle furnace at AOAC 942.05 (ignition at 600°C for 2 h). The moisture content was determined by weight loss upon drying at 105°C for 9 h. The total carbohydrate content was determined using an AA3 Continuous Flow Analyzer (Bran+Luebbe, Norderstedt, Germany) as described elsewhere (Moorby et al., 2006).

### Fourier Transform Infrared Spectrum Analysis

Fourier-transform infrared (FT-IR) spectroscopy of Taisui TS-2007S exopolymers, xanthan gum, and PVA was performed using the KBr pressed disk technique and a FT-IR spectrometer (Nicolet IS10, Thermo Scientific, Waltham, MA, United States) from 400 to 4,000 cm^−1^ and at a resolution of 4 cm^−1^. Freeze-dried powder of TS-2007S exopolymers (2 mg), xanthan gum (2 mg), or PVA (2 mg) was grounded with 80 mg KBr in an agate mortar for 10 min, after which the disks were pressed. Twenty scans were collected and averaged for each sample and background to obtain the spectrum.

### NMR Analysis

The NMR spectra of Taisui TS-2007S exopolymers, xanthan gum, and PVA were recorded on a Bruker AV 400 spectrometer at 400.13 MHz for ^1^H and 100.78 MHz for ^13^C.

### Monosaccharide Composition Analysis

Reversed-phase-high-performance liquid chromatography (RP-HPLC) precolumn derivatization with 1-phenyl-3-methyl-5-pyrazolone (PMP; Dai et al., 2010) was used to determine the monosaccharide composition in Taisui TS-2007S exopolymers. In brief, TS-2007S exopolymers were hydrolyzed with trifluoroacetic acid, after which the resulting monosaccharides were derivatized with PMP, and the monosaccharide-PMP derivatives were then tested by HPLC (Agilent 1100, Agilent Technologies, Palo Alto, CA, United States) using an RP-C18 HPLC column (250 mm length, 4.6 mm i.d., and 5 μm particle size; Agilent, CA, United States).

### DNA Extraction and High-Throughput Sequencing

DNA was extracted from Taisui TS-2007S using the CTAB method (Stewart and Via, 1993). The bacterial V3–V4 region of the 16S rRNA gene was amplified using two rounds of PCR. The PCR primers used in the first round were 341F [CCTACACGACGCTCTTCCGATCTN (barcode) CCTACGGGNGGCWGCAG] and 805R [CCTACACGACGCTCTTCCGATCTN (barcode) GYGCASCAGKCGMGAAW], while the PCR primers used in the second round were Illumina bridge PCR-compatible primers. The archaeal V3–V4 16S rRNA gene region was amplified using three rounds of PCR. The PCR primers used in the first round were M-340F (CCCTAYGGGGYGCASCAG) and GU1ST-1000R (GGCCATGCACYWCYTCTC); the PCR primers used in the second round were the Illumina MiSeq sequencing platform V3–V4 universal primers 349F [CCTACACGACGCTCTTCCGATCTN (barcode) GYGCASCAGKCGMGAAW] and 806R (GACTGGAGTTCCTTGGCACCCGAGAATTCCAGGACTACVSGGGTATCTAAT); and the PCR primers used in the third round were Illumina bridge PCR-compatible primers. The fungal ITS1–2 region was amplified using two rounds of PCR. The PCR primers used in the first round were ITS1F [CCCTACACGACGCTCTTCCGATCTN (barcode) CTTGGTCATTTAGAGGAAGTAA] and ITS2R (GTGACTGGAGTTCCTTGGCACCCGAGAATTCCAGCTGCGTTCTTCATCGATGC), while those used in the second round were Illumina bridge PCR-compatible primers.

High-throughput sequencing of PCR amplicons was conducted on an Illumina MiSeq platform by Sangon Biotech Co., Ltd. (Shanghai, China). High-quality sequence reads were clustered into operational taxonomic units (OTUs) at a 97% similarity level. The remaining OTUs were taxonomically classified using BLASTn-based searches in the Ribosomal Database Project (RDP) and SILVA databases with a similarity threshold of 97% using UPARSE (version 7.1), with chimeric sequences identified and removed using UCHIME. The metagenomic functional composition prediction analysis using bacterial and archaeal 16S data in the latest Clusters of Orthologous Groups of proteins (COG) database was performed using the PICRUSt pipeline as described elsewhere (Langille et al., 2013; Zhang et al., 2018).

### Data Availability

The raw sequence data reported in the present study have been deposited in the Genome Sequence Archive (Wang et al., 2017) in the Beijing Institute of Genomics (BIG) Data Center (Partners, 2020), Chinese Academy of Sciences, under the accession number CRA003090 and is publicly accessible at https://bigd.big.ac.cn/gsa.

## Results

### Chemical Composition of Taisui TS-2007S

**Figure 2:**
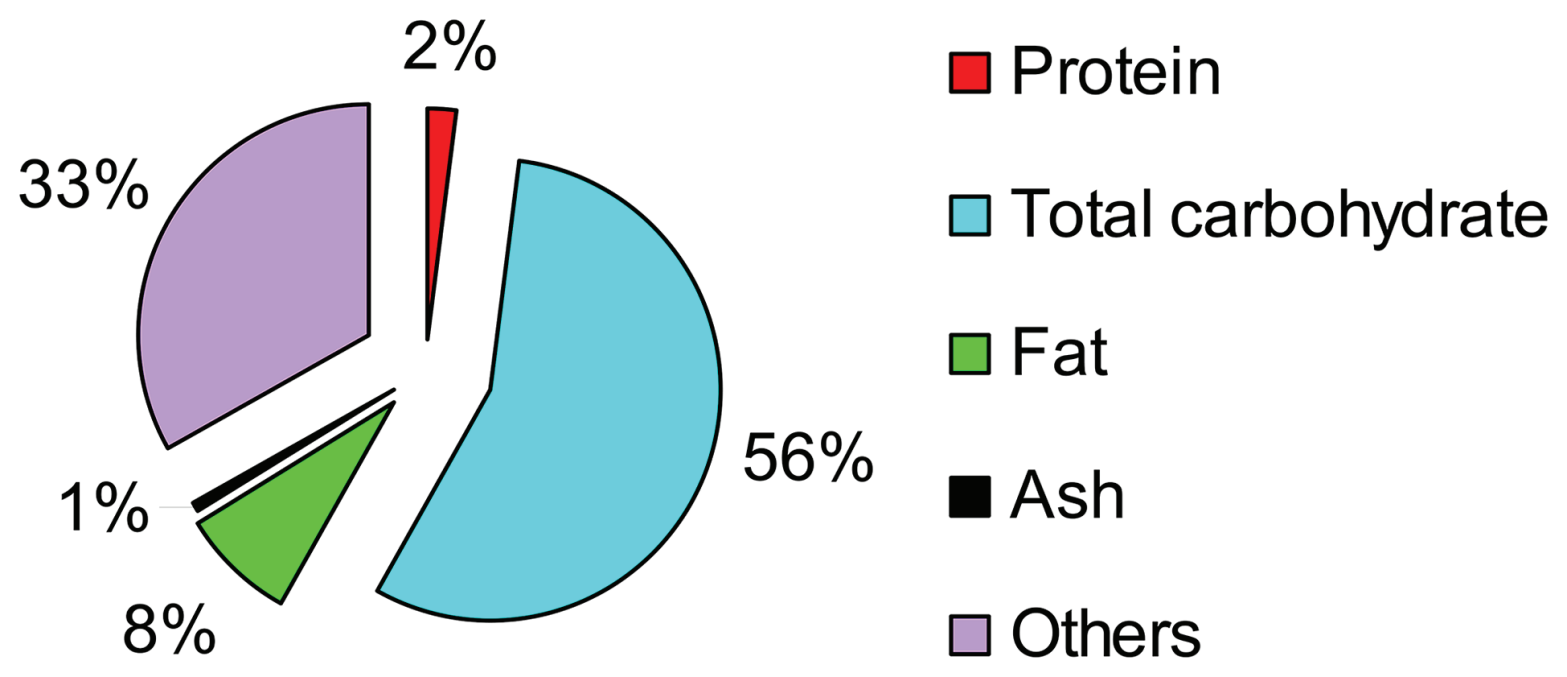
Analysis result of the chemical composition in Taisui TS-2007S on dry weight.

The primary component in Taisui TS-2007S was identified as carbohydrate, followed by fat. The measured protein content in Taisui TS-2007S was low, at only 2% dry weight (Figure 2).

### FT-IR Spectroscopy of Taisui TS-2007S Exopolymers and Selected Polymers

**Figure 3:**
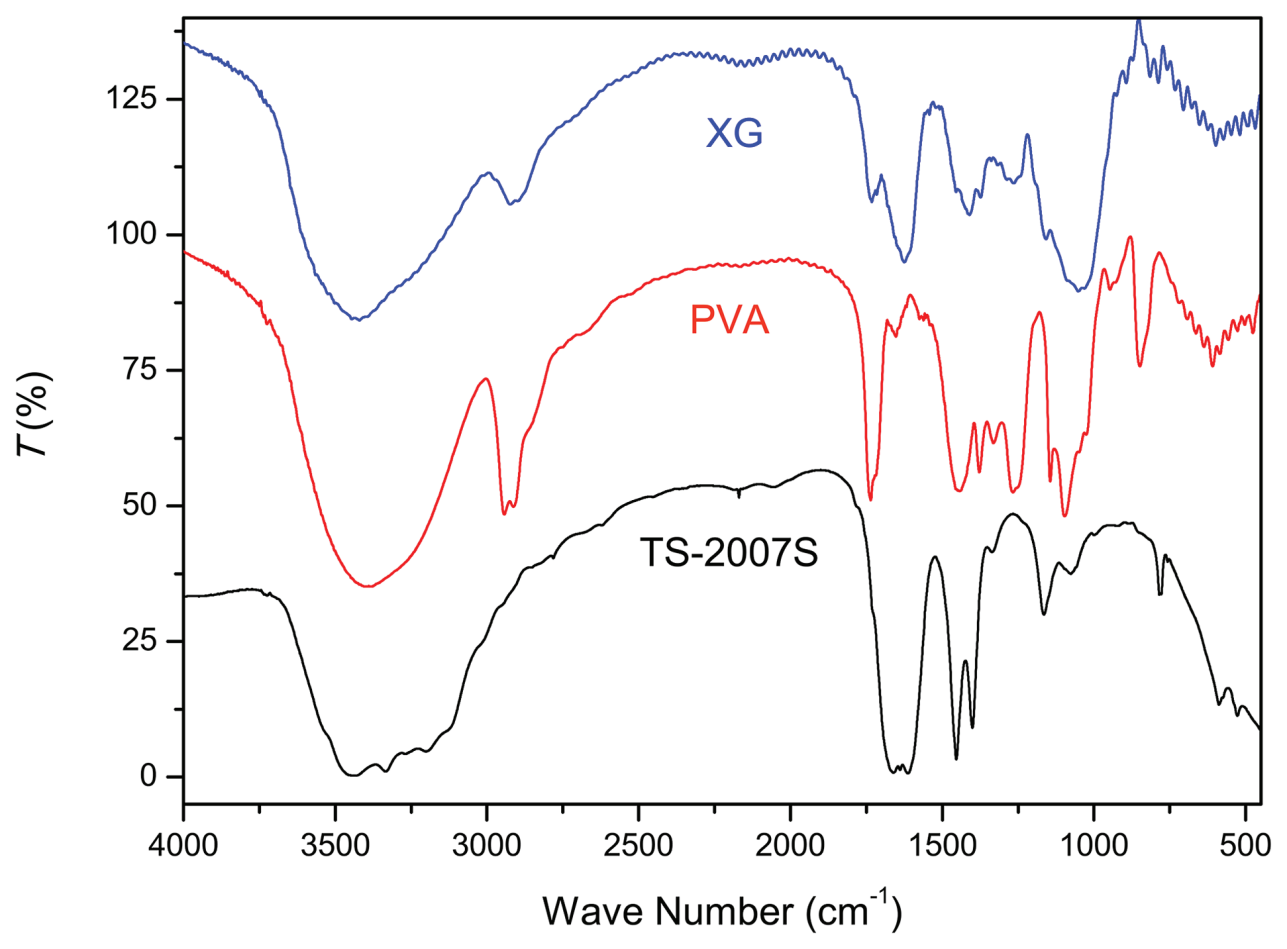
Fourier-transform infrared (FT-IR) spectroscopy of Taisui TS-2007S exopolymers, xanthan gum, and polyvinyl alcohol (PVA). PVA, polyvinyl alcohol; XG, xanthan gum.

The FT-IR spectra of TS-2007S exopolymers, xanthan gum, and PVA were analyzed from 400 to 4,000 cm^−1^. The absorption peaks at 3,440 cm^−1^ for all of the three polymers were ascribed to O-H stretching vibration in xanthan gum and PVA. However, the width of this peak was wider in TS-2007S than in xanthan gum and PVA, indicating that the intensity of hydroxyl association in TS-2007S was higher than that observed in xanthan gum and PVA. The peak at 2,930 cm^−1^ in xanthan gum and PVA was attributed to C-H stretching vibration, and was not observed in TS-2007S. Peaks at 1,661 and 1,611 cm^−1^ in TS-2007S and xanthan gum were attributed to C=O asymmetric stretching vibration in xanthan gum, which occurred at peaks at 1,762 and 1,653 cm^−1^ in PVA. Peaks at 1,454, 1,400, and 1,338 cm^−1^ in TS-2007S and xanthan gum were attributed to C-H variable-angle vibration in xanthan gum, while peaks at 1,443, 1,377, and 1,331 cm^−1^ in PVA were attributed to C-H stretching and bending. Peaks at 1164 and 1,078 cm^−1^ in TS-2007S and xanthan gum were the characteristic of C-O-C pyranose ring asymmetric vibration in xanthan gum, whereas absorption peaks at 1,144 and 1,097 cm^−1^ were ascribed to C-O stretching vibration for PVA (Mansur et al., 2008; Wang et al., 2010; Korbag and Mohamed Saleh, 2016; Kang et al., 2019; Figure 3).

### NMR Analysis of Taisui TS-2007S Exopolymers and Selected Polymers

**Figure 4:**
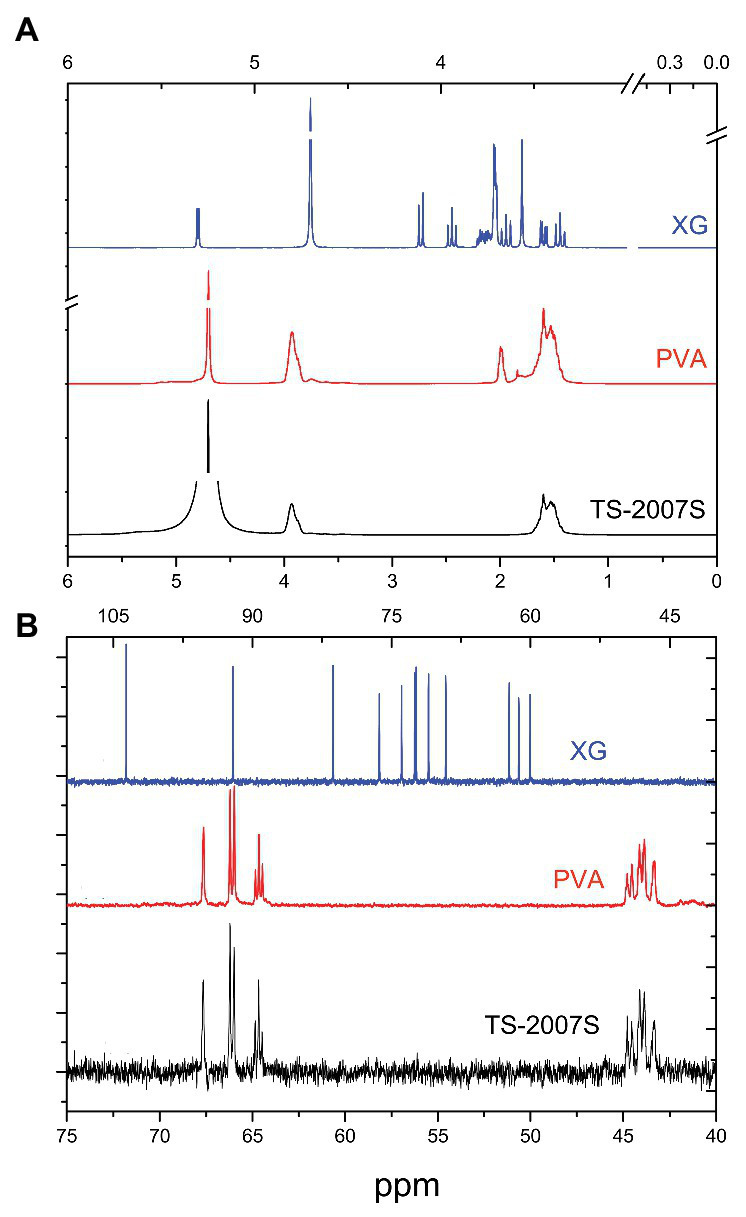
NMR spectra of TS-2007S exopolymers, xanthan gum, and PVA in D_2_O. **(A)** ^1^H NMR spectra; **(B)** ^13^C NMR spectra. PVA, polyvinyl alcohol; XG, xanthan gum.

The ^1^H NMR spectra of TS-2007S exopolymers, xanthan gum, and PVA in D_2_O showed peaks at 3.8–4.0 and 1.4–1.7 ppm for HOH and CH_2_ hydrogens in PVA (Gippert and Brown, 1984), while the glucuronic acid of hexose or pentose in xanthan gum was recorded as peaks at 2.1–2.7 ppm (Faria et al., 2011; Figure 4A). The ^13^C NMR spectrum of TS-2007S exopolymers in D_2_O was similar to that of PVA but differed from xanthan gum (Figure 4B). Unlike PVA, the NMR spectra of TS-2007S exopolymers and xanthan gum in dimethyl sulfoxide (DMSO) were not determined because TS-2007S exopolymers and xanthan gum were not soluble in DMSO.

### Monosaccharide Composition of Taisui TS-2007S Exopolymers

**Figure 5:**
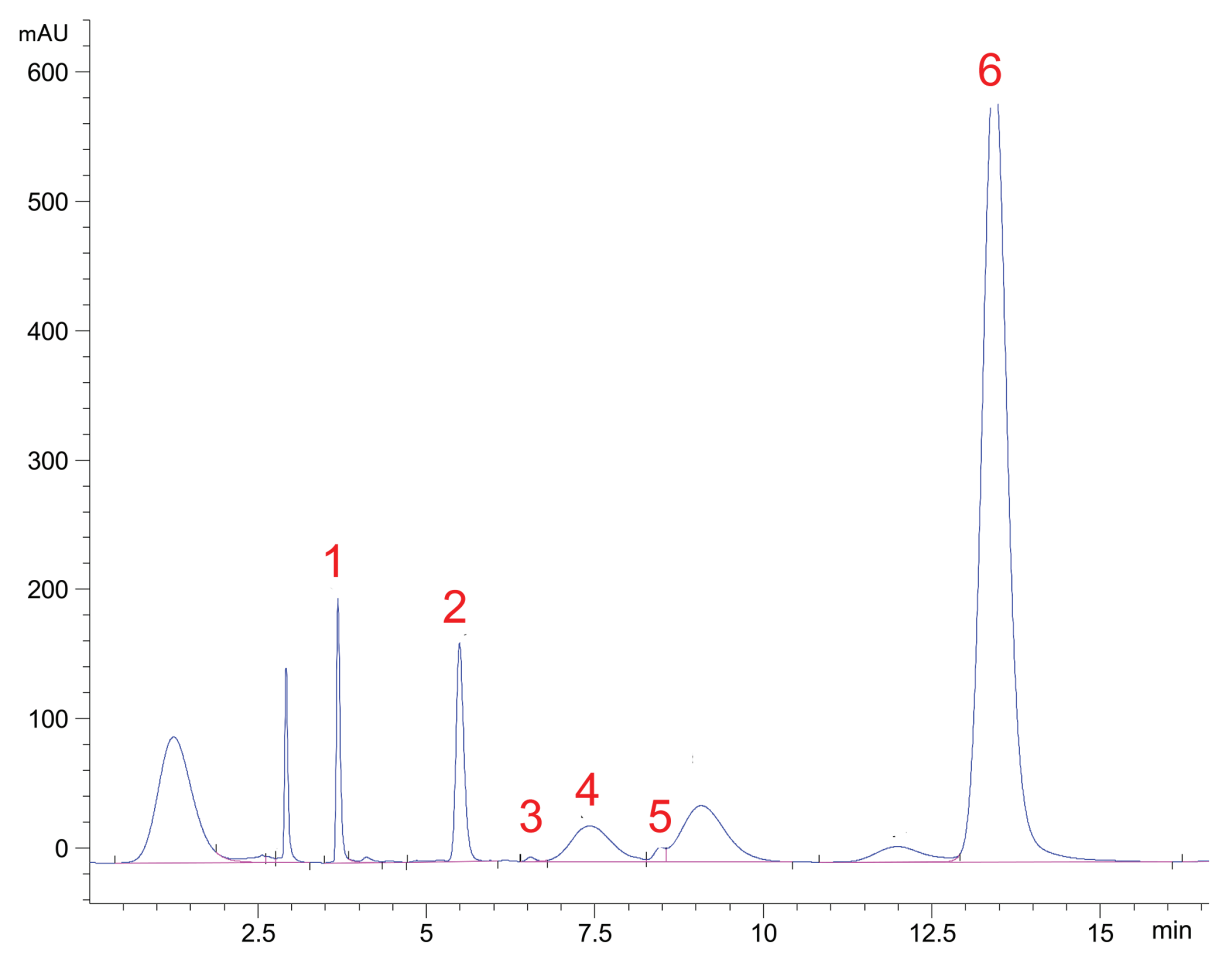
High-performance liquid chromatography (HPLC) chromatograms of 1-phenyl-3-methyl-5-pyrazolone (PMP) derivatives of component monosaccharides released from Taisui TS-2007S exopolymers. Peaks: 1, Man, mannose; 2, Rha, rhamnose; 3, GA, glucuronic acid; 4, Gal, galactose; 5, Xyl, xylose; 6, Fuc, fucose.

High-performance liquid chromatography analysis of the monosaccharide composition of TS-2007S exopolymers showed that TS-2007S exopolymers were primarily composed of fucose, rhamnose, mannose, and glucuronic acid at a molecular ratio of 87.90:7.49:4.45:0.15. TS-2007S exopolymers also contained trace amounts of glucuronic acid, galactose, and xylose (Figure 5).

### Evaluation of the Microbial Community Structure and its Functional Profiling in Taisui TS-2007S

**Figure 6:**
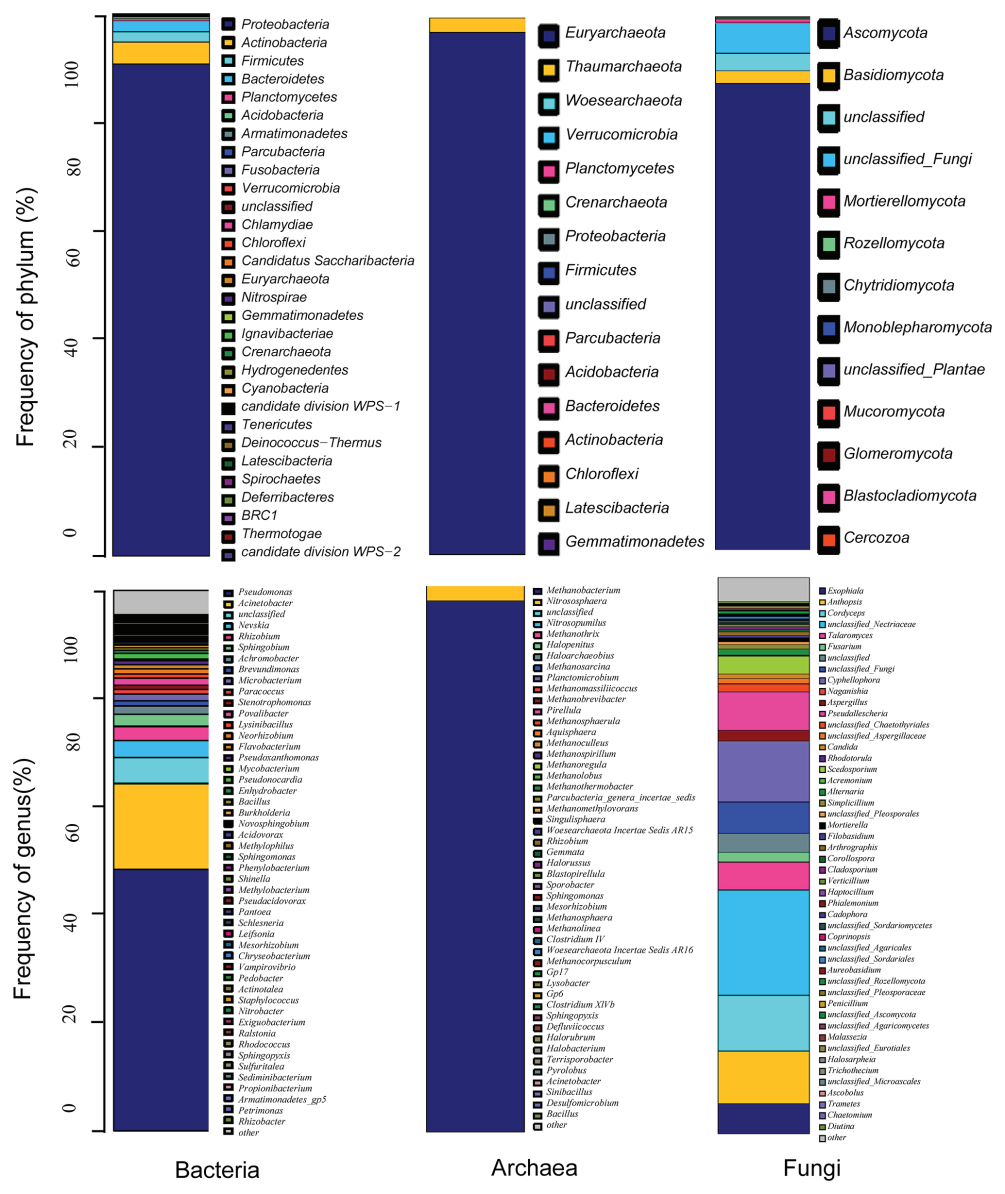
Microbial community structure of Taisui TS-2007S. Distribution of microbe in TS-2007S at phylum and genus levels.

From the metagenomic data of Taisui TS-2007S, 46,676 reads were identified as bacterial and were clustered into 1,095 OTUs. All bacterial OTUs belonged to 17 phyla, with the dominant phyla (relative abundance > 1%) including Proteobacteria (90.77%), Actinobacteria (4.06%), Bacteroidetes (2.01%), and Firmicutes (1.89%). The structure and distribution of the bacterial community was also analyzed at the genus level. Ten genera were identified as dominant genera (relative abundance > 1%), which contributed to 82.3% of the read abundance. Of these dominant genera, the predominant genera were *Pseudomonas* (48.32%), *Acinetobacter* (15.92%), *Nevskia* (3.09%), and *Rhizobium* (2.66%). Photosynthetic bacteria and sulfate-reducing bacteria (autotrophic bacteria) were almost absent (Figure 6).

**Figure 7:**
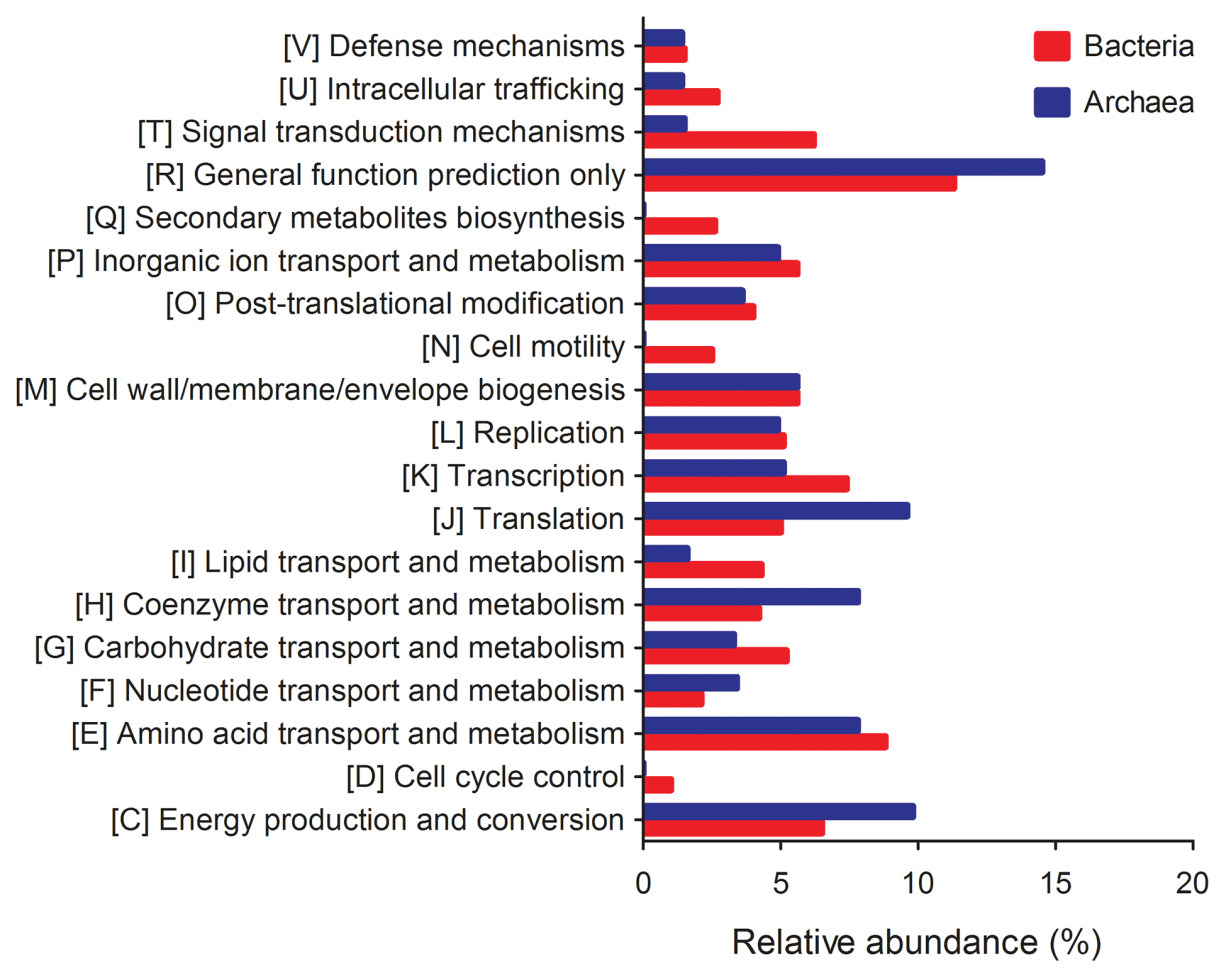
The relative abundance of predictive functional genes of bacterial and archaeal community in Taisui TS-2007S. Genes with unknown functions and <1% in abundance were not included.

The metagenomic functional composition of TS-2007S was predicted by PICRUSt, in which bacterial genes related to quorum sensing, phosphodiesterase, cell motility, adhesion, and polysaccharide synthesis/transport contributed to biofilm formation (Castiblanco and Sundin, 2016; Rana et al., 2020), while those encoding protein-degrading enzymes (protease and peptidase), polysaccharide or oligosaccharide-degrading enzymes (cellulase, glucosidase, xylosidase, and glucuronidase), lipid-degrading enzymes (esterase and lipase), phosphomonoesterases (phosphatase), and oxidoreductases (peroxidase) were involved in biofilm degradation (Flemming and Wingender, 2010). The genes related to cell motility showed the highest abundance reaching up to 2.450% among the genes involved in biofilm formation. One quorum sensing gene, four phosphodiesterase genes, three adhesion genes, and five polysaccharide synthesis/transport genes with abundances of >0.01% were identified as being related to biofilm formation and were divided into multiple COG functional categories, with the total abundances of these groups of genes being 0.024, 0.050, 0.123, and 0.178%, respectively. Among the genes involved in biofilm degradation, those encoding protease, peptidase, phosphatase, and esterase/lipase enzymes were more abundant, with 19, 22, 22, and 7 genes detected with total abundances of 0.796, 0.857, 0.795, and 0.334%, respectively. Only 1, 2, 2, 1, and 3 genes encoding cellulase, glucosidase, xylosidase, glucuronidase and peroxidase enzymes were detected with total abundances of 0.013, 0.076, 0.029, 0.020, and 0.100%, respectively (Figure 7, Supplementary Table S1).

A total of 63,185 sequenced reads of the archaeal 16S rRNA gene were obtained from Taisui TS-2007S that reads clustered into 236 OTUs. Three archaeal genera were identified, including *Methanobacterium* (relative abundance 97.15%), *Nitrososphaera* (relative abundance 2.79%), and *Methanothrix* (relative abundance 0.02%), which belong to the phyla Euryarchaeota, Thaumarchaeota, and Crenarchaeota, respectively. Additionally, 0.03% of the reads were unclassified (Figure 6).

The archaeal metagenome-based gene functional abundance data of TS-2007S showed that only 0.407% of the genes were related to cell motility, and genes involved in quorum sensing, phosphodiesterase activity, and adhesion were absent among the gene related to biofilm formation. Regarding genes encoding protease, peptidase and phosphatase enzymes involved in biofilm degradation, 8, 5, and 12 genes were present at >0.01% in abundance and had total abundances of 1.074, 0.447, and 0.887%, respectively, whereas cellulase and esterase/lipase-encoding genes were each represented by only 1 gene and had an abundance of 0.062% (Figure 7, Supplementary Table S2).

The fungal ITS1–2 region was sequenced to generate 81,285 reads and was clustered into 1,060 OTUs in Taisui TS-2007S. The OTUs comprised nine phyla and 103 genera. The most abundant phyla were Ascomycota and Basidiomycota, which contributed 87.43 and 2.36% of the read abundance, respectively. The dominant genera (relative abundance > 1%) were unclassified_*Nectriaceae* (18.92%), *Cyphellophora* (10.91%), *Cordyceps* (10.12%), *Anthopsis* (9.46%), *Pseudallescheria* (6.82%), *Exophiala* (5.39%), *Talaromyces* (5.01%), *Scedosporium* (3.22%), *Aspergillus* (1.97%), *Fusarium* (1.75%), unclassified_*Chaetothyriales* (1.51%), *Alternaria* (1.05%), and unclassified_*Aspergillaceae* (1.01%; Figure 6).

## Discussion

Taisuis have been previously proposed to comprise huge slime mold and fungi complexes, myxobacteria, or fungi alone (Li et al., 2020). However, Taisuis have extremely low nitrogen and protein contents, much lower than those present in living creatures, and should not be considered to be solely biological in nature (Zhao et al., 2018; Li et al., 2020). The results of the present study also showed that the Taisui TS-2007S protein content is much lower than that observed in microorganisms, the crude protein content of which is greater than 30% (Matassa et al., 2016). Recently, several groups used FT-IR, NMR, gel permeation chromatography (GPC), and AMS-^14^C measurements to examine Taisuis and observed that many Taisui samples from different locations in China and with different textures and colors were PVA of abiotic origin that was over 40,000 years old (Zheng and Dong, 2010; Zhao et al., 2018; Li et al., 2020). These results raise the question as to whether PVA can be naturally generated.

PVA is an artificial polymer first synthesized in 1924 by Hermann and Haehnel by saponifying poly (vinyl ester) with sodium hydroxide solution, resulting in a PVA solution. This PVA solution was essentially made from polyvinyl acetate, not the polymerization of vinyl alcohol, because vinyl alcohol is a highly unstable monomer relative to its tautomer acetaldehyde. Furthermore, the vinyl ester monomer precursor is poisonous to the coordination catalyst, making PVA difficult to obtain (Halima, 2016; Huang et al., 2020). Taken together, these factors indicate that PVA should not be naturally formed. However, PVA is easily degradable by microorganisms (Solaro et al., 2000) and is hard to preserve, unless the synthesis rate exceeds the degradation rate. In the present study, we observed that the FT-IR spectra of Taisui TS-2007S exopolymers were distinctly different from those of PVA but similar to those of xanthan gum. Although TS-2007S exopolymers yielded an NMR spectrum similar to that of PVA and different from xanthan gum, neither TS-2007S exopolymers nor xanthan gum had the organic solvent solubility of PVA. Therefore, Taisui is mostly not PVA of abiotic origin that formed tens of thousands of years ago.

The principal component of Taisui TS-2007S is carbohydrates, with the protein, fat, and mineral contents that are extremely low. The carbohydrates were primarily comprised fucose, rhamnose, mannose, and glucuronic acid at a molar ratio 87.90:7.49:4.45:0.15 and were mostly the extracellular polymeric substances (EPS) synthesized by the Taisui builder microorganisms. This is the first report of such a high level of fucose-containing extracellular polysaccharides, which is similar to the cell wall polysaccharides of some brown alga (Marais and Joseleau, 2001; Zvyagintseva et al., 2003). Although bacteria, fungi, and microalgae all produce fucose-rich extracellular polysaccharides, the monosaccharide molar ratio of fucose in bacterial extracellular polysaccharides, especially from the many members of the family *Enterobacteriaceae*, is much higher than that of fungi and microalgae. These include Clavan, which is produced by *Clavibacter michiganensis* and is composed of D-glucose, D-galactose, L-fucose, and pyruvic acid at a molar ratio of 1:1:2:1, and extracellular polysaccharide, which is produced by the marine bacterium *Enterobacter cloacae* and is composed of fucose, galactose, glucose, and glucuronic acid at a molar ratio of 2:1:1:1 (Roca et al., 2015). FucoPol, synthesized by *Enterobacter* A47, is composed of fucose, galactose, glucose, and glucuronic acid at a molar ratio of 3.1:2.3:1.9:1.0 (Antunes et al., 2017). Many *Pseudomonas* and *Acinetobacter* strains secrete fucose-rich exopolysaccharide (Ghods et al., 2015; Cavallero et al., 2020; Knirel et al., 2020), and Taisui TS-2007S EPS may be produced by its predominant bacterial members *Pseudomonas* and *Acinetobacter*. The high-fucose molar ratio in Taisui TS-2007S EPS may result from the metabolism of easily degradable sugars, other than fucose, by heterotrophic microorganisms in the microbial mat.

Fucose is a rare sugar, and fucose and fucose-containing polysaccharides, fucose-containing oligosaccharides, glycoproteins, and glycolipids have a variety of biological activities, particularly immunomodulation (Vetvicka and Vetvickova, 2017), antioxidant (Su and Li, 2020), anticoagulant (Springer et al., 1957), anti-inflammatory (Pomin, 2015), antiviral (Mandal et al., 2007), antitumor (Oliveira et al., 2017), and anticancer activities (Nunes and Coimbra, 2019). Thus, these activities may be the mechanism of therapeutic activity for Taisuis described in ancient Chinese medical books.

The predominant bacterial phylum in Taisui TS-2007S was identified as Proteobacteria (relative abundance 90.77%), with the predominant genera including *Pseudomonas* (48.32%) and *Acinetobacter* (15.92%). The predominant archaeal phylum and dominant genus in Taisui TS-2007S were Euryarchaeota and *Methanobacterium*, with a relative abundance of 97.15%. The predominant fungal phylum in Taisui TS-2007S was Ascomycota (relative abundance 87.43%), and the predominant genera were *Cyphellophora* (10.91%), *Cordyceps* (10.12%), and *Anthopsis* (9.46%). However, no microbial phyla or genera with such high levels of abundance have been observed in water and soil (Gilbert et al., 2009; Carvajal et al., 2016; Ma et al., 2020). The microbial community structure of Taisui TS-2007S was obviously different from that in the surrounding environment. Therefore, the microbes that resided in TS-2007S were not simply embedded from the environment.

*Pseudomonas* and *Acinetobacter* are common bacteria inhabiting soil and water (Zhang et al., 2019a; Wang et al., 2020a) and can be typically form biofilms (Flemming and Wingender, 2010; Rana et al., 2020). Importantly, *Pseudomonas* is a metabolically versatile, ubiquitous heterotroph with broad catabolic and transport capabilities (Bonilla-Rosso et al., 2012). *Pseudomonas fluorescens* species are aerobes; but some strains can use nitrate as an electron acceptor instead of oxygen (Silby et al., 2009). *Pseudomonas* strains have large genomes, high affinity transporters, and broad sensing capabilities that allow them to adapt and grow in nutrient-rich environments (Lauro et al., 2009). Nevertheless, some *Pseudomonas* strains can use several carbon sources at very low concentrations (van der Kooij et al., 1982) and can form biofilms under starvation conditions to maximize exposure to diluted nutrients (Kroukamp et al., 2010). Many *Pseudomonas* strains secrete carboxylic acids to efficiently solubilize mineralized inorganic phosphates (Fankem et al., 2008; Woo et al., 2010). Thus, *Pseudomonas* should be capable of generating the EPS and driving the geochemical cycle in the Taisui. Photoautotrophic bacteria and chemical autotrophic bacteria were not detected in the TS-2007S metagenome, possibly due to this Taisui having resided underground for a long period of time.

*Methanobacterium* species are the overwhelmingly dominant methanogenic archaea in the active layer of alpine grasslands (Wang et al., 2020b) and are the dominant archaeal genera in hot spring microbial mats (Ward et al., 1990). *Methanobacterium* species can produce methane by using H_2_ and CO_2_ (Conrad and Klose, 2006), providing an organic carbon source for methanotrophic bacteria and archaea in the Taisui.

Extracellular polymeric substance plays an important role in protecting cells (Zhang et al., 2019b, 2020) and generates the framework of bacterial mats (Krüger et al., 2008). The bacteria and archaea harbored in TS-2007S possessed many genes associated with EPS formation, protein-degrading enzyme, and phosphomonoesterase, but few genes encoding polysaccharide or oligosaccharide-degrading enzyme. Thus, there was a little degradation and utilization of the EPS by bacteria and archaea residing in TS-2007S. In another study, lower grazing animals in water or soil do not harbor L-fucose dehydrogenase and therefore cannot utilize fucose-rich polysaccharides (Bakke and Kajiyama, 2004). Thus, Taisui EPS may be conserved.

The microbial community structure of TS-2007S was shown to be composed of heterotrophic microorganisms, and there was a little influence of photosynthesis on carbonate mineral precipitation. The local freshwater and soil had a low carbonate mineral content, and the EPS matrix of TS-2007S probably did not bind tightly with calcium, potentially explaining why Taisui TS-2007S was not lithified.

In summary, the unidentified biological object Taisui TS-2007S that was discovered in soil in China was characterized in the present study. Its exopolymers could potentially be microbial EPS, and its microbial community structure differed from that in the local water and soil environments. Based on these results, we propose that Taisui TS-2007S should be considered a microbial mat formed in soil that was discovered by accident.

## Data Availability Statement

The original contributions presented in the study are included in the article/Supplementary Material), further inquiries can be directed to the corresponding authors.

## Author Contributions

TS, CW, and LQ designed the research. TS, HL, CZ, and DS performed the lab work. TS and HL analyzed and visualized the data. TS, CW, and LQ wrote and edited the manuscript. All authors contributed to the article and approved the submitted version.

### Conflict of Interest

The authors declare that the research was conducted in the absence of any commercial or financial relationships that could be construed as a potential conflict of interest.
