# Supplementary material for: Taisui TS-2007S, a Large Microbial Mat Discovered in Soil in China
Source: Front Microbiol. 2020 Nov 11;11:592034. doi: 10.3389/fmicb.2020.592034 (PMC7690426; doi:10.3389/fmicb.2020.592034)
Supplement: Supplementary file 1 [file Data_Sheet_1.docx]

**
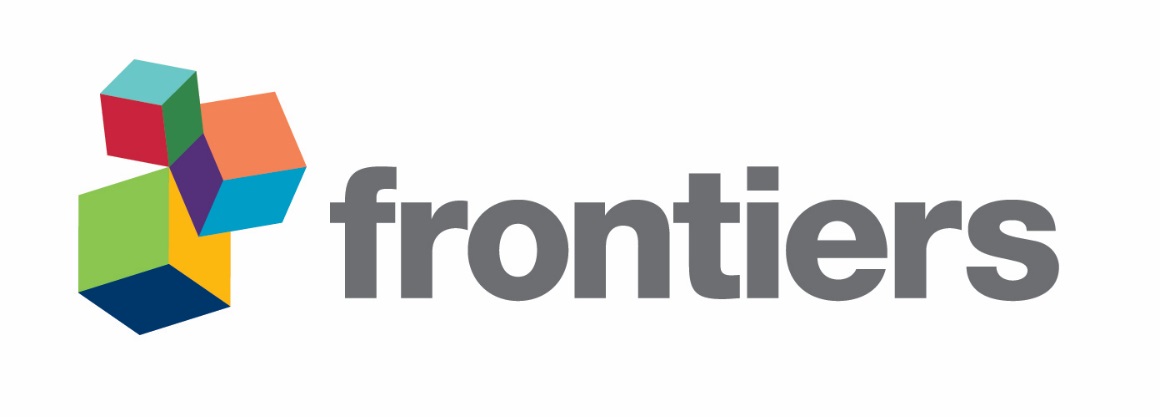
**

**Supplementary Table 1.** Relative abundance of predictive specific bacterial genes for biofilm formation and degradation in Taisui TS-2007S. Genes with unknown functions and <0.01% in abundance were not included. COG category references Figure 7.

| Function | COG number | COG category | Relative abundance (%) | COG name |
| --- | --- | --- | --- | --- |
| Quorum sensing | COG1551 | T | 0.024 | Carbon storage regulator (could also regulate swarming and quorum sensing) |
| Phosphodiesterase | COG0737 | F | 0.027 | 5'-nucleotidase/2',3'-cyclic phosphodiesterase and related esterases |
|  | COG3540 | P | 0.022 | Phosphodiesterase/alkaline phosphatase D |
|  | COG1182 | I | 0.019 | Acyl carrier protein phosphodiesterase |
|  | COG0584 | C | 0.055 | Glycerophosphoryl diester phosphodiesterase |
| Adhesion | COG4531 | P | 0.018 | ABC-type Zn2+ transport system, periplasmic component/surface adhesin |
|  | COG0803 | P | 0.014 | ABC-type metal ion transport system, periplasmic component/surface adhesin |
|  | COG3419 | N | 0.019 | Tfp pilus assembly protein, tip-associated adhesin PilY1 |
| Polysaccharide synthesis and transport | COG1596 | M | 0.039 | Periplasmic protein involved in polysaccharide export |
|  | COG1134 | G | 0.015 | ABC-type polysaccharide/polyol phosphate transport system, ATPase component |
|  | COG2148 | M | 0.044 | Sugar transferases involved in lipopolysaccharide synthesis |
|  | COG1208 | M | 0.035 | Nucleoside-diphosphate-sugar pyrophosphorylase involved in lipopolysaccharide biosynthesis/translation initiation factor 2B |
|  | COG1682 | G | 0.044 | ABC-type polysaccharide/polyol phosphate export systems, permease component |
| Protease | COG0330 | O | 0.090 | Membrane protease subunits, stomatin/prohibitin homologs |
|  | COG0533 | O | 0.029 | Metal-dependent proteases with possible chaperone activity |
|  | COG1305 | E | 0.074 | Transglutaminase-like enzymes, putative cysteine proteases |
|  | COG0826 | O | 0.048 | Collagenase and related proteases |
|  | COG0793 | M | 0.057 | Periplasmic protease |
|  | COG0740 | O | 0.050 | Protease subunit of ATP-dependent Clp proteases |
|  | COG1770 | E | 0.019 | Protease II |
|  | COG1585 | O | 0.025 | Membrane protein implicated in regulation of membrane protease activity |
|  | COG0465 | O | 0.046 | ATP-dependent Zn proteases |
|  | COG0265 | O | 0.081 | Trypsin-like serine proteases, typically periplasmic, contain C-terminal PDZ domain |
|  | COG1030 | O | 0.016 | Membrane-bound serine protease (ClpP class) |
|  | COG0616 | O | 0.050 | Periplasmic serine proteases (ClpP class) |
|  | COG4618 | R | 0.010 | ABC-type protease/lipase transport system, ATPase and permease components |
|  | COG1220 | O | 0.022 | ATP-dependent protease HslVU (ClpYQ), ATPase subunit |
|  | COG1404 | O | 0.030 | Subtilisin-like serine proteases |
|  | COG5405 | O | 0.022 | ATP-dependent protease HslVU (ClpYQ), peptidase subunit |
|  | COG0501 | O | 0.055 | Zn-dependent protease with chaperone function |
|  | COG0466 | O | 0.041 | ATP-dependent Lon protease, bacterial type |
|  | COG1219 | O | 0.030 | ATP-dependent protease Clp, ATPase subunit |
| Peptidase | COG4942 | D | 0.029 | Membrane-bound metallopeptidase |
|  | COG0739 | M | 0.119 | Membrane proteins related to metalloendopeptidases |
|  | COG0744 | M | 0.059 | Membrane carboxypeptidase (penicillin-binding protein) |
|  | COG2355 | E | 0.024 | Zn-dependent dipeptidase, microsomal dipeptidase homolog |
|  | COG3770 | M | 0.003 | Murein endopeptidase |
|  | COG0260 | E | 0.037 | Leucyl aminopeptidase |
|  | COG0339 | E | 0.032 | Zn-dependent oligopeptidases |
|  | COG0024 | J | 0.038 | Methionine aminopeptidase |
|  | COG2027 | M | 0.020 | D-alanyl-D-alanine carboxypeptidase (penicillin-binding protein 4) |
|  | COG0308 | E | 0.039 | Aminopeptidase N |
|  | COG1974 | K | 0.047 | SOS-response transcriptional repressors (RecA-mediated autopeptidases) |
|  | COG1686 | M | 0.054 | D-alanyl-D-alanine carboxypeptidase |
|  | COG5009 | M | 0.028 | Membrane carboxypeptidase/penicillin-binding protein |
|  | COG1989 | N | 0.027 | Type II secretory pathway, prepilin signal peptidase PulO and related peptidases |
|  | COG1362 | E | 0.012 | Aspartyl aminopeptidase |
|  | COG0597 | M | 0.044 | Lipoprotein signal peptidase |
|  | COG1506 | E | 0.084 | Dipeptidyl aminopeptidases/acylaminoacyl-peptidases |
|  | COG0006 | E | 0.063 | Xaa-Pro aminopeptidase |
|  | COG4242 | Q | 0.014 | Cyanophycinase and related exopeptidases |
|  | COG2274 | V | 0.034 | ABC-type bacteriocin/lantibiotic exporters, contain an N-terminal double-glycine peptidase domain |
|  | COG1876 | M | 0.005 | D-alanyl-D-alanine carboxypeptidase |
|  | COG1025 | O | 0.012 | Secreted/periplasmic Zn-dependent peptidases, insulinase-like |
|  | COG0681 | U | 0.034 | Signal peptidase I |
| Cellulase | COG1363 | G | 0.013 | Cellulase M and related proteins |
| Glucosidase | COG2723 | G | 0.023 | Beta-glucosidase/6-phospho-beta-glucosidase/beta-galactosidase |
|  | COG1472 | G | 0.052 | Beta-glucosidase-related glycosidases |
| Xylosidase | COG3664 | G | 0.013 | Beta-xylosidase |
|  | COG3507 | G | 0.016 | Beta-xylosidase |
| Glucuronidase | COG3250 | G | 0.020 | Beta-galactosidase/beta-glucuronidase |
| Esterase/lipase | COG0657 | I | 0.090 | Esterase/lipase |
|  | COG2267 | I | 0.085 | Lysophospholipase |
|  | COG5380 | O | 0.015 | Lipase chaperone |
|  | COG2755 | E | 0.078 | Lysophospholipase L1 and related esterases |
|  | COG2829 | M | 0.018 | Outer membrane phospholipase A |
|  | COG2272 | I | 0.012 | Carboxylesterase type B |
|  | COG1946 | I | 0.037 | Acyl-CoA thioesterase |
| Phosphatase | COG0560 | E | 0.081 | Phosphoserine phosphatase |
|  | COG1368 | M | 0.021 | Phosphoglycerol transferase and related proteins, alkaline phosphatase superfamily |
|  | COG1218 | P | 0.023 | 3'-Phosphoadenosine 5'-phosphosulfate (PAPS) 3'-phosphatase |
|  | COG3808 | C | 0.010 | Inorganic pyrophosphatase |
|  | COG0127 | F | 0.029 | Xanthosine triphosphate pyrophosphatase |
|  | COG0631 | T | 0.044 | Serine/threonine protein phosphatase |
|  | COG2062 | T | 0.030 | Phosphohistidine phosphatase SixA |
|  | COG2208 | T | 0.041 | Serine phosphatase RsbU, regulator of sigma subunit |
|  | COG0394 | T | 0.063 | Protein-tyrosine-phosphatase |
|  | COG1267 | I | 0.020 | Phosphatidylglycerophosphatase A and related proteins |
|  | COG1254 | C | 0.010 | Acylphosphatases |
|  | COG0671 | I | 0.019 | Membrane-associated phospholipid phosphatase |
|  | COG0248 | F | 0.035 | Exopolyphosphatase |
|  | COG0241 | E | 0.021 | Histidinol phosphatase and related phosphatases |
|  | COG0406 | G | 0.071 | Fructose-2,6-bisphosphatase |
|  | COG4579 | T | 0.014 | Isocitrate dehydrogenase kinase/phosphatase |
|  | COG1051 | F | 0.119 | ADP-ribose pyrophosphatase |
|  | COG2365 | T | 0.012 | Protein tyrosine/serine phosphatase |
|  | COG0158 | G | 0.024 | Fructose-1,6-bisphosphatase |
|  | COG0301 | H | 0.012 | Thiamine biosynthesis ATP pyrophosphatase |
|  | COG0221 | C | 0.030 | Inorganic pyrophosphatase |
|  | COG0483 | G | 0.068 | Archaeal fructose-1,6-bisphosphatase and related enzymes of inositol monophosphatase family |
| Peroxidase | COG0386 | O | 0.051 | Glutathione peroxidase |
|  | COG1858 | P | 0.025 | Cytochrome c peroxidase |
|  | COG0376 | P | 0.024 | Catalase (peroxidase I) |

**Supplementary Table 2.** Relative abundance of predictive specific archaeal genes for biofilm degradation in Taisui TS-2007S. Genes with unknown functions and <0.01% in abundance were not included. COG category references Figure 7.

| Function | COG number | COG category | Relative abundance (%) | COG name |
| --- | --- | --- | --- | --- |
| Protease | COG4870 | O | 0.187 | Cysteine protease |
|  | COG0501 | O | 0.128 | Zn-dependent protease with chaperone function |
|  | COG0466 | O | 0.062 | ATP-dependent Lon protease, bacterial type |
|  | COG0330 | O | 0.066 | Membrane protease subunits, stomatin/prohibitin homologs |
|  | COG0533 | J | 0.066 | Metal-dependent proteases with possible chaperone activity |
|  | COG1305 | O | 0.249 | Transglutaminase-like enzymes, putative cysteine proteases |
|  | COG0826 | O | 0.187 | Collagenase and related proteases |
|  | COG1994 | O | 0.128 | Zn-dependent proteases |
| Peptidase | COG3764 | M | 0.125 | Sortase (surface protein transpeptidase) |
|  | COG0024 | J | 0.066 | Methionine aminopeptidase |
|  | COG0006 | E | 0.070 | Xaa-Pro aminopeptidase |
|  | COG2274 | V | 0.125 | ABC-type bacteriocin/lantibiotic exporters, contain an N-terminal double-glycine peptidase domain |
|  | COG1506 | E | 0.062 | Dipeptidyl aminopeptidases/acylaminoacyl-peptidases |
| Cellulase | COG1363 | E | 0.062 | Cellulase M and related proteins |
| Esterase/lipase | COG0657 | I | 0.062 | Esterase/lipase |
| Phosphatase | COG1387 | E | 0.062 | Histidinol phosphatase and related hydrolases of the PHP family |
|  | COG0394 | T | 0.062 | Protein-tyrosine-phosphatase |
|  | COG1267 | I | 0.062 | Phosphatidylglycerophosphatase A and related proteins |
|  | COG0671 | I | 0.062 | Membrane-associated phospholipid phosphatase |
|  | COG0248 | F | 0.062 | Exopolyphosphatase |
|  | COG1051 | F | 0.191 | ADP-ribose pyrophosphatase |
|  | COG0301 | H | 0.066 | Thiamine biosynthesis ATP pyrophosphatase |
|  | COG0221 | C | 0.062 | Inorganic pyrophosphatase |
|  | COG0483 | G | 0.062 | Archaeal fructose-1,6-bisphosphatase and related enzymes of inositol monophosphatase family |
|  | COG1980 | G | 0.066 | Archaeal fructose 1,6-bisphosphatase |
|  | COG0560 | E | 0.062 | Phosphoserine phosphatase |
|  | COG0127 | F | 0.066 | Xanthosine triphosphate pyrophosphatase |
